# Supplementary material for: Nitrogen-Doped Hierarchical Porous Carbon Derived from Coal for High-Performance Supercapacitor
Source: Molecules. 2023 Apr 23;28(9):3660. doi: 10.3390/molecules28093660 (PMC10180139; doi:10.3390/molecules28093660)
Supplement: Supplementary file 1 [file molecules-28-03660-s001.zip › molecules-2335129-supplementary.pdf]

Supplementary Materials

Nitrogen-Doped Hierarchical Porous Carbon Derived from Coal for High-Performance Supercapacitor

Leiming Cai, Yanzhe Zhang, Rui Ma, Xia Feng, Lihua Yan, Dianzeng Jia, Mengjiao Xu, Lili Ai, Nannan Guo * and Luxiang Wang *

State Key Laboratory of Chemistry and Utilization of Carbon Based Energy Resources, College of Chemistry, Xinjiang University, Urumqi 830017, China

* Correspondence: guonan067@xju.edu.cn (N.G.); wangluxiang@xju.edu.cn (L.W.)

Table S1. Pore structures of all samples.

Sample	BET SSA (m^2 g^{-1})	Pore Volume (cm^3 g^{-1})	Mesopore Volume (cm^3 g^{-1})	Micropore Volume (cm^3 g^{-1})	$V_{micro}/(V_{micro} + V_{meso})$
Mi-CPC	1268	0.56	0.12	0.44	78.6%
h-CPC	1735	1.27	0.76	0.51	40%
Me-CPC	320	0.17	0.07	0.10	59%

Notes: The pore size distribution is calculated from the corresponding adsorption branch of N_2 isotherm by the nonlocal density functional theory (NLDFT) method for micropores and Barrett-Joyner-Halenda method for mesopores. The total pore volume here is equal to the sum of mesopore volume and micropore volume.

Table S2. elemental analysis of the raw coal, OC and prepared samples.

Sample	C (wt.%)	H (wt.%)	N (wt.%)	O (wt.%)
Coal	77.29	4.13	0.93	17.65
OC	48.11	1.83	4.2	45.86
Mi-CPC	70.4	0.55	1.71	28.34
h-CPC	75.98	0.55	2.87	20.6
Me-CPC	65.99	0.3	10.93	22.78

Table S3. Elements content obtained from the XPS data of all samples.

Samples	C (at. %)	N (at. %)	O (at. %)
Mi-CPC	90.91	1.40	7.69
h-CPC	85.03	5.34	9.63
Me-CPC	74.76	18.31	6.93

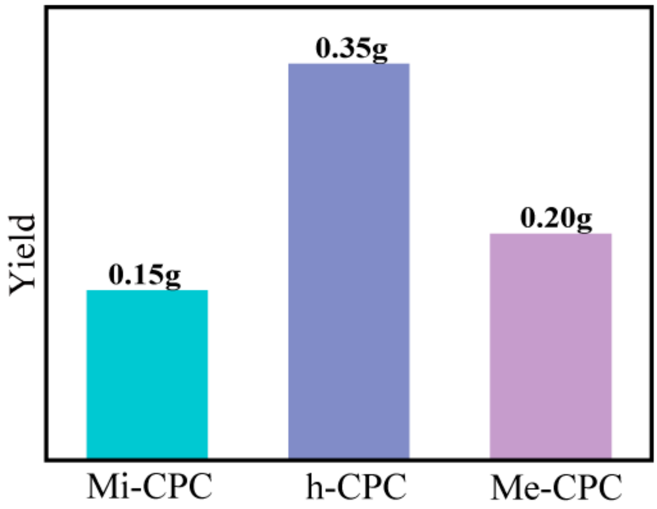

Figure S1. The yield data of all samples.

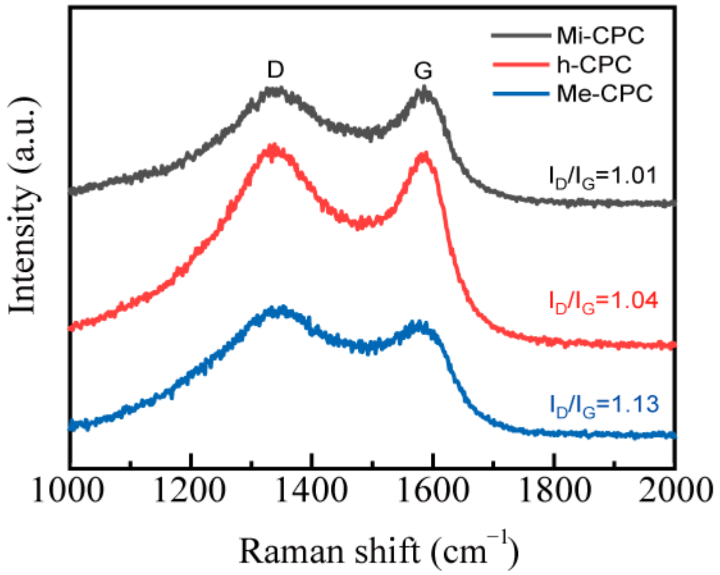

Figure S2. The Raman spectra of all samples.

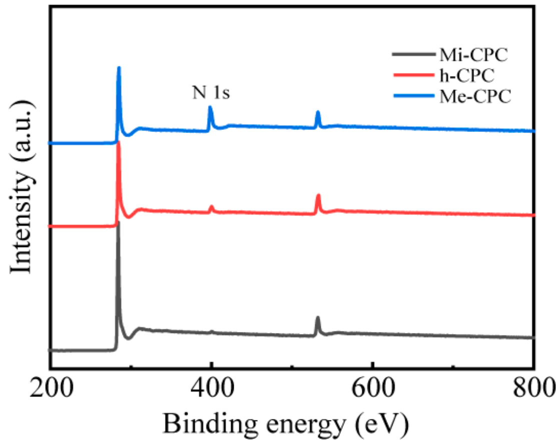

Figure S3. XPS survey spectra of Mi-CPC, h-CPC and Me-CPC.

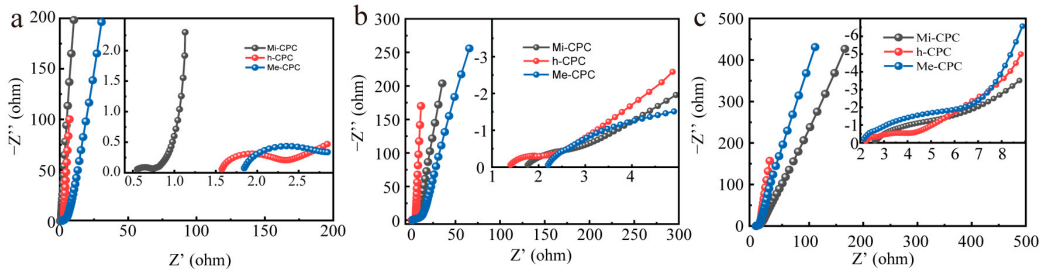

Figure S4. Nyquist plots of all resultant samples electrodes: (a) in two-electrode system with 6 M KOH electrolyte, (b) in two-electrode system with 1 M Na_2SO_4 electrolyte, (c) in two-electrode system with EMIM BF_4 electrolyte.

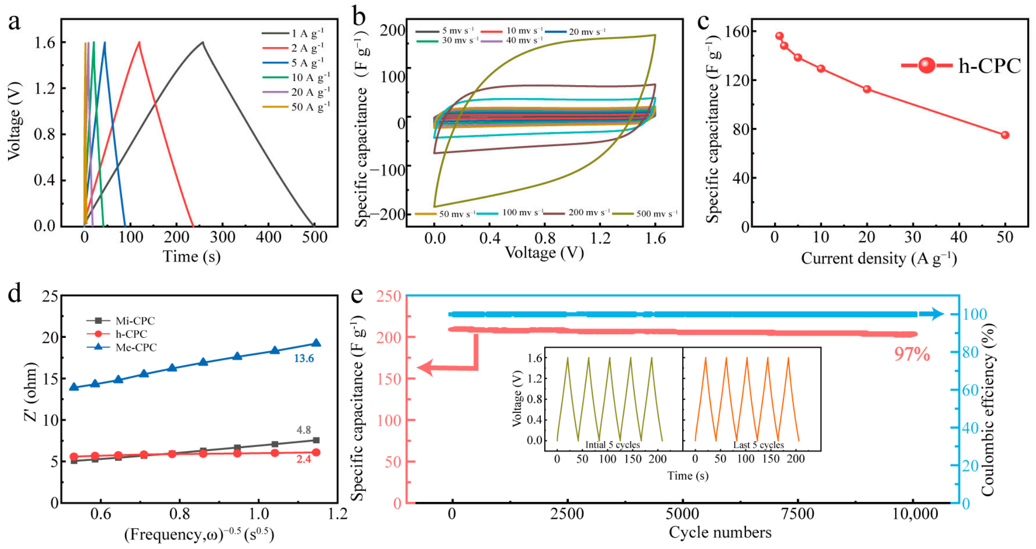

Figure S5. Electrochemical performance of h-CPC in a 1 M Na_2SO_4 electrolyte for a two-electrode system: (a) GCD curves, (b) CV curves, (c) specific capacitance at different current densities. (d) Z′ versus $\omega^{-1/2}$ plots for Mi-CPC, h-CPC and Me-CPC. and (e) cycling performance of h-CPC.

Table S4. Comparisons of the capacitive performances for the h-CPC electrode in this work and reported carbon materials from literatures.

Sample	Energy Density/ Power Density ($Wh\ kg^{-1}/W\ kg^{-1}$)	Electrolyte	Ref.
N, S self-doped porous carbon nanosheets	7.4/486.7	6M KOH	1
Porous carbon nanospheres	6.5/80<br>3.7/4000	6M KOH	2
N,O-codoped egg-box-like carbons	8.09/257	6M KOH	3
Rice husk-based hierarchical porous carbon	7.7/241	6M KOH	4
Hollow N-doped carbon frameworks	4.36/249	6M KOH	5
N-doped porous carbon nanosheets	7.15/50.4	6M KOH	6
Hierarchical porous carbon spheres	7.8/31.2<br>5.1/6200	6M KOH	7
Heteroatom incorporated porous carbon	9/227	6M KOH	8
Hierarchical porous carbon nanorods	6.77/100<br>3.6/10000	6M KOH	9
Hierarchical coal-based porous carbon materials	8.3/250	6M KOH	This work
Honeycomblike mesoporous carbons	31.7/620<br>18/10600	1 M $TEABF_4$/AN	10
N-doped graphene	34.5/679.4<br>12.8/251400	$BMIMBF_4$	11
Natural shiitake mushroom	41/875<br>31/6750	1 M ($TEABF_4$/AN)	12
Hair derived micro/mesoporous carbon	45.33/75<br>29/2243	1 M $LiPF_6$ EC/DEC	13
Graphitic carbon nanosheets	40/750<br>19/20000	BMPY TFSI	14
Hierarchical coal-based porous carbon materials	48.3/759	$BMIMBF_4$	This work

References

[1] Miao, L.; Zhu, D.; Liu, M.; Duan, H.; Wang, Z.; Lv, Y.; Xiong, W.; Zhu, Q.; Li, L.; Chai, X.; et al. Cooking carbon with protic salt: Nitrogen and sulfur self-doped porous carbon nanosheets for supercapacitors. *Chem. Eng. J.* **2018**, *347*, 233–242.

[2] Song, Z.; Zhu, D.; Li, L.; Chen, T.; Duan, H.; Wang, Z.; Lv, Y.; Xiong, W.; Liu, M.; Gan, L. Ultrahigh energy density of a N, O codoped carbon nanosphere based all-solid-state symmetric supercapacitor. *J. Mater. Chem. A* **2019**, *7*, 1177–1186.

[3] Wei, F.; He, X.; Ma, L.; Zhang, H.; Xiao, N.; Qiu, J. 3D N, O-Codoped Egg-Box-Like Carbons with Tuned Channels for High Areal Capacitance Supercapacitors. *Nano-Micro Lett.* **2020**, *12*, 82.

[4] Chen, Z.; Wang, X.; Xue, B.; Li, W.; Ding, Z.; Yang, X.; Qiu, J.; Wang, Z. Rice husk-based hierarchical porous carbon for high performance supercapacitors: The structure-performance relationship. *Carbon* **2020**, *161*, 432–444.

[5] Wang, M.; Yang, J.; Jia, K.; Liu, S.; Hu, C.; Qiu, J. Boosting Supercapacitor Performance of Graphene by Coupling with Nitro-gen-Doped Hollow Carbon Frameworks. *Chem. Eur. J.* **2020**, *26*, 2897–2903.

[6] Wang, D.; Wang, Y.; Chen, Y.; Liu, W.; Wang, H.; Zhao, P.; Li, Y.; Zhang, J.; Dong, Y.; Hu, S.; et al. Coal tar pitch derived N-doped porous carbon nanosheets by the in-situ formed g-C_3N_4 as a template for supercapacitor electrodes. *Electrochim. Acta* **2018**, *283*, 132–140.

[7] Pang, J.; Zhang, W.; Zhang, H.; Zhang, J.; Zhang, H.; Cao, G.; Han, M.; Yang, Y. Sustainable nitrogen-containing hierarchical porous carbon spheres derived

from sodium lignosulfonate for high-performance supercapacitors. *Carbon* **2018**, *132*, 280–293.

[8] Jia, H.; Sun, J.; Xie, X.; Yin, K.; Sun, L. Cicada slough-derived heteroatom incorporated porous carbon for supercapacitor: Ultra-high gravimetric capacitance. *Carbon* **2019**, *143*, 309–317.

[9] Fang, L.; Xie, Y.; Wang, Y.; Zhang, Z.; Liu, P.; Cheng, N.; Liu, J.; Tu, Y.; Zhao, H.; Zhang, J. Facile synthesis of hierarchical porous carbon nanorods for supercapacitors application. *Appl. Surf. Sci.* **2019**, *464*, 479–487.

[10] Guan, T.; Zhao, J.; Zhang, G.; Wang, J.; Zhang, D.; Li, K. Template-Free Synthesis of Honeycomblike Porous Carbon Rich in Specific 2–5 nm Mesopores from a Pitch-Based Polymer for a High-Performance Supercapacitor. *ACS Sustain. Chem. Eng.* **2019**, *7*, 2116–2126.

[11] Dai, S.; Liu, Z.; Zhao, B.; Zeng, J.; Hu, H.; Zhang, Q.; Chen, D.; Qu, C.; Dang, D.; Liu, M. A high-performance supercapacitor electrode based on N-doped porous graphene. *J. Power Sources* **2018**, *387*, 43–48.

[12] Cheng, P.; Gao, S.; Zang, P.; Yang, X.; Bai, Y.; Xu, H.; Liu, Z.; Lei, Z. Hierarchically porous carbon by activation of shiitake mushroom for capacitive energy storage. *Carbon* **2015**, *93*, 315–324.

[13] Qian, W.; Sun, F.; Xu, Y.; Qiu, L.; Liu, C.; Wang, S.; Yan, F. Human hair-derived carbon flakes for electrochemical supercapacitors. *Energy Environ. Sci.* **2014**, *7*, 379–386.

[14] Wang, H.; Xu, Z.; Kohandehghan, A.; Li, Z.; Cui, K.; Tan, X.; Stephenson, T.J.; King'ondu, C.K.; Holt, C.M.B.; Olsen, B.C.; et al. Interconnected Carbon Nanosheets Derived from Hemp for Ultrafast Supercapacitors with High Energy. *ACS Nano* **2013**, *7*, 5131–5141.
